# Is pseudomyopia associated with anxiety and related disorders?

**DOI:** 10.12669/pjms.37.5.3991

**Published:** 2021

**Authors:** Halil Kara, Erdogan Yasar, Ugur Gurlevik

**Affiliations:** 1 Halil Kara, MD. Assistant Professor, Department of Child and Adolescent Psychiatry, Aksaray Education and Research Hospital, Aksaray University, Faculty of Medicine, Aksaray, Turkey; 2 Erdogan Yasar, MD. Assistant Professor, Department of Ophthalmology, Aksaray Education and Research Hospital, Aksaray University, Faculty of Medicine, Aksaray, Turkey; 3 Ugur Gurlevik, MD. Assistant Professor, Department of Ophthalmology, Aksaray Education and Research Hospital, Aksaray University, Faculty of Medicine, Aksaray, Turkey

**Keywords:** Accomodative spasm, Anxiety, Pseudomyopia, Psychiatric disorders

## Abstract

**Objectives:**

To investigate in detail the exact relationship between Pseudomyopia, also termed accommodative spasm, and psychiatric disorders.

**Methods:**

Twenty-one young people between the ages of 12-18 who were diagnosed with pseudomyopia between March 2019 and July 2020 in the ophthalmology eye clinic of a university hospital, Turkey were included in the study. A difference of at least 2.20 D between refractive error measurements before and after cycloplegic drop was accepted as pseudomyopia. Scl-90-r symptom screening scale was applied to each case. Afterwards, each case was evaluated by k-sads-pl-dsm-5-t semi-structured technique according to age. The relationship between psychiatric disorders in cases of pseudomyopia was examined.

**Results:**

The average age of patients in the study was 15,4 ± 1,9 (12-18), 13 (61,9%) girl and 8 (38,1%) boy. The mean initial refraction was -4,19D ± 2,48D (-1,75D /-8,50D), and the result refraction was +0,38D ± 0,22D (0,25D / -1,00D). The average amount of accommodation was 4,56D ± 2,59D (2,25D / 9,50D). Following the SCL-90-R screening scale and psychiatric evaluation, five generalized anxiety disorders, three obsessive compulsive disorders, three panic disorders, one social anxiety disorder, one posttraumatic stress disorder, one conversion disorder, one major depressive disorder were diagnosed. As a result, 15 (71,4%) of 21 patients were treated with a psychiatric diagnosis. In addition, a positive correlation (p: 0,010-r: 0,621, p: 0,029-r: 0,546) was detected between anxiety- somatization scores and accommodation amount.

**Conclusions:**

It is necessary to request psychiatric consultation in each case of pseudomyopia. Comorbidity of anxiety and depressive disorders is more common in pseudomyopia cases. In addition, as the severity of psychiatric symptoms increases, the amount of accommodation also appears to increase.

## INTRODUCTION

Pseudomyopia is the result of excessive accommodation and its also known as accommodative spasm. When eye wants to view distance targets or focusses for near, it adjusts appropriate contracture of the ciliary muscle and zonules to reflect light on the fovea. The reflex, occurred during near view, consists of three components; accommodation, convergence and miosis.^1^ A spasm during near reflex will lead to excessive constriction of the lens and reflect the image in front of the retina, thus distorting the vision. Therefore, excessive accommodation will produce a pseudomyopia and cause a blurred vision, which is called as pseudomyopia, or accommodative spasm. In such a case, the patient will complain of sudden blurred vision, diplopia, limited ocular motility, ocular pain and photophobia, which are the results of obvious myopic refractive error. Signs and symptoms are episodic and often expected to disappear by cycloplegia. However, it has been hypothesized that accommodative spasm (AS), convergence spasm (CS) and spasm of near reflex (SNR) are the presence of a single disease in different clinical manifestations or diseases in the SNR spectrum.^2^ Besides, some authors make a distinction between these spasms in certain situations. For example, London and colleagues reported that treatment and prognosis for AS and CS due to head trauma are different and should be considered as a clinical case different from SNR.^3^

Head trauma appears to be the most common cause of near reflex spasm encountered in the studies of etiology. Psychiatric disorders such as anxiety disorders and personality disorders are reported as being the second most common cause.^4^-^6^ Neurological diseases such as multiple sclerosis, posterior fossa anomalies and pituitary tumors are also involved in the etiology.^7^,^8^ In this study, we aimed to investigate the relationship between pseudomyopia and psychiatric diseases in detail.

## METHODS

Twenty-one right eyes of twenty-one patients with accommodative spasm were included in this study. All the procedures were carried out in accordance with the Helsinki Declaration. Ethics Committee approval was obtained from the local Ethics Committee from Human Research Ethics Committee of Aksaray University (Number: 2019/12-11) as well as written informed consent from the parents of all children prior to participation. After obtaining ethical approval for the study of Aksaray/Turkey is in the eye clinic of a university hospital, the diagnosis is made pseudomyopia between March 2019-July 2020 those children and adolescents.

###  Exclusion criteria

It included patients with strabismus or amblyopia, presence of eye diseases (corneal, lens, macular, retinal disease) that could affect refractive error, presence of ocular infection, history of ocular and refractive surgery, and history of systemic disease or drug use that could affect refraction.

Demographic questioning was performed for all patients. Cycloplegic refraction measurement (with 1% cyclopentolate) was applied to patients who had occasional blurred vision complaints and who were diagnosed with myopia through autorefractometer (TONOREF™ III, Nidek Co. Ltd. Japan), At least 2,20D difference between the measurements before and after the cycloplegic drop was considered as accommodative spasm (pseudomyopia).^5^,^6^ The next day, visual acuity was examined through Snellen. Psychiatric consultation was conducted for the patients who were diagnosed with pseudomyopia. Scl-90-r symptom screening scale was applied to each case, which was then evaluated with psychiatric aspect by k-sads-pl-dsm-5-t semi-structured technique according to age. The patients were diagnosed as a result of the examination. Medical or cognitive behavioral therapy treatment was planned for the cases that were deemed necessary. However, the measurement tools used are listed below.

### Socio-demographic data form

The sociodemographic characteristics of the patients included in the study were obtained by using semi-structured sociodemographic data form created by the researchers. In the form, gender, age, educational status, age of parents, previous psychiatric diagnosis and treatment, medical history of disorders, degree of kinship, economic status, presence of siblings and number of siblings were questioned along with head trauma, unconsciousness, hospitalization history, preschool education and duration, family history of chronic disease and family history of psychiatric disorders.

### Symptom Scan List (SCl-90-R)

Based on the theoretical propositions regarding the role of schemas in the development of psychopathology, this scale was developed by Derogatis and used for the purpose of examining the combined validity. Turkish validity and reliability studies were conducted by Dag.^9^ This scale, as a psychiatric screening tool to measure the level of difficulty or negative stress response of the individual in addition to psychological and physical symptoms, consist of 90 items based on a 5-point (no / very low / moderate / very high / severe) Likert-type assessment. It is based on self- declaration. 9 sub-scales reflecting 9 different symptom groups are as follows: Somatization, Obsessive-Compulsive, Interpersonal Sensitivity, Depression, Anxiety, Hostility, Phobic Anxiety, Paranoid Thought and Psychoticism. The scale also has three indexes, General Symptom Level (GSI), Positive Symptom Total (PST), and Positive Symptom Level (PSDI), and an additional scale consisting of items evaluating feelings of guilt, eating and sleeping problems. Studies related to the original and Turkish form of the scale indicate that the scale is valid and reliable, so it has been used in many studies in Turkey.^10^ SCL 90-R is a test developed for psychological symptom screening. The parameters not diagnosed with a disease, but with a severity score above one are considered to be high, whereas parameters with a severity score above 2,5 are considered pathological. However, for parameters between 1-2,5, the person clinically needs a psychiatric evaluation.

### Schedule for Affective Disorders and Schizophrenia for School-Age Children-Present and Lifetime Version, DSM-5 (K-SADS-PL-DSM-5-T) Scans

Many mental disorders except learning difficulties, developmental disorders and schizophrenia with negative symptoms.^11^ The Turkish validity and reliability test of the K-SADS-PL-DSM-5-T was performed by Gokler and his peers in 2016.^12^ In this sample, the patients are evaluated by individual interview and parents’ interview. Psychiatric diagnosis was diagnosed by a child and adolescent psychiatrist for patients who met the diagnostic criteria according to DSM-5. KD-SADS were used at these interviews to assist the clinician in diagnosing.

### Statistical analysis

Statistical analysis was performed using SPSS 23.0 (SPSS Inc., Chicago, IL). The normality of the data distribution was evaluated by the Shapiro-Wilk test and found to be normal. Student t-test was used to compare the averages of the observed values of a variable in two different conditions. Descriptive statistical analysis method was used along with Pearson correlation analysis to estimate the linear relationship between variables. Statistical significance was accepted as p <0.05.

## RESULTS

The average age of the patients included in the study was 15.4 ± 1.9 (min: 12-max:18), of which 13 (61.9%) were female and 8 (38.1%) were male. (Table-I). The average initial refraction values were detected as mean -4.19D ± SD 2.48D (min: -1.75D /max: -8.50D) and the result refraction values mean + 0.38D ± SD 0.22D (min: 0.25D / max:1.00D). The mean amount of accommodation was 4.56D ± SD 2.59D (min: 2.25D / max: 9.5D) (Table-II).

**Table-I:** Age values of patients.

	Female(n:13)	Male (n:8)	t	P value
Age (years)	16.01 ± SD 2.80	14.7 ± SD 2.44	1.85^a^	0.82

**Table-II:** Refraction values of patients.

	Initial refraction (mean values)	Resultant refraction (mean values)	Accommodation values (mean values)
Mean refraction values	-4.19D ± SD 2.48D	+ 0.38D ± SD 0.22D	4.56D ± SD 2.59D

D: dioptri; ^a^: Student t-test, SD: standard deviation

According to Symptom Screening List (Scl-90-R) results, the average symptom severity scores were determined as follows: Somatization 1.20 ± 0.25 (min: 0- max:2), Obsessive Compulsive 1.27 ± 0.62 (min:0.20- max: 2.10), Interpersonal Sensitivity 1.37 ± 0.68 (min:0.17- max:2.19), Depression 1.09 ± 0.71 (min:0.33- max:2.37), Anxiety 1.80 ± 0.36 (min:0.50- max:2.77), Anger-Enmity 0.78 ± 0.45 (min:0- max:1.17), Phobia 1.11 ± 0.67 (min:0- max:2), Paranoid Special 0.77 ±0.50 (min:0- max:1.11), Psychoticism 0.24 ± 0.26 (min:0- max:0.77), Supplementary Scale 1.21 ± 0.72 (min:0- max:2.10), Overall Score 1.08.

**Fig.1:**
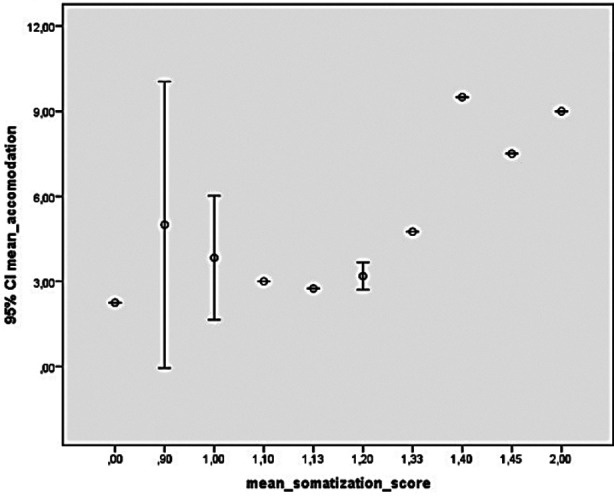
Correlation analysis between mean accommodation and mean somatization score. This figure shows that mean diopter measurement correlates with the mean somatization subset score in the scl-90-r test. As the somatization score increase, the measured myopia severity increase.

**Fig.2:**
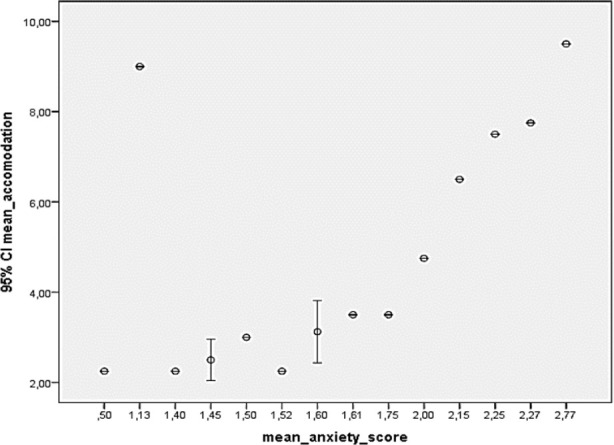
Correlation analysis between mean accommodation and mean anxiety score. This figure shows that mean diopter measurement correlates with the mean anxiety score in the scl-90-r test. As the anxiety score increase, the measured myopia severity increase.

The result of the correlation analysis between the average score of the symptoms in the SCL-90-R screening list and the average accommodation amount was detected as follows: Moderate positive correlation significant with somatization (p: 0,029, r: 0.546) and moderate positive correlation with significant anxiety (p: 0.010, r:0.621). This correlation is shown in Fig.1 and 2. No significant correlation was found between the average scores of other symptoms in the screening list and the average accommodation amount (p> 0.05).

Each patient who underwent SCL-90-R screening scale was evaluated with K-SADS-PL. In conclusion, 5 patients were diagnosed with General Anxiety Disorder (GAD), 3 with Obsessive-Compulsive Disorder (OCD), 3 with Panic Disorder (PD), 1 with Social Anxiety Disorder (SAD), 1 with Post-Traumatic Stress Disorder (PTSD), 1 with Conversion Disorder (CD) and 1 patient with Major Depressive Disorder (MDD). Other patients had subclinical anxious symptoms. In other words, 15 (71.4%) of 21 patients were treated with a psychiatric diagnosis and medical or cognitive behavioral therapy.

## DISCUSSION

Etiology is the most important factor for determining treatment response in pseudomyopia. Coexistence of psychiatric diseases in pseudomyopia patients has been reported in previous studies.^4^-^6^ In addition, there is a study suggesting that all patients diagnosed with pseudomyopia should be evaluated psychiatrically regardless of the etiology.^13^ Notably, some patients with etiology of head trauma, even when they are diagnosed with pseudomyopia, are reported to have internalizing problems and increased anxiety. In addition, pseudomyopia caused by a psychiatric factor can be considered as a better condition with respect to pseudomyopia caused by other factors.^14^

In our study, pseudomyopia was more common in women than men. This may be related to the higher prevalence of anxiety disorders in women.^15^ In the study of Shetty et al., It was stated that pseudomyopia was observed more frequently in women.^16^ We found psychiatric disease comorbidity 71.4% in our study. The prevalence of anxiety disorders in the normal population is 7.3%.^17^ This situation made us think that there may be a relationship between anxiety disorders, depression and pseudomyopia. Notably, there was a positive correlation between average somatization – anxiety symptoms scores and average accommodation amounts. It suggested that there is a positive significant relationship between the severity of psychiatric disease and the severity of pseudomyopia. Our study is perhaps the first study in the literature investigating the relationship between psychiatric diseases in pseudomyopia. However, many authors who do not suggest an etiologic cause for pseudomyopia suggest that the presence of a psychiatric entity is the most likely cause.^1^ However, there are studies stating that pseudomyopia is more common in children.^19^ Our study is compatible with these data.

When we examine etiologically, common brain regions affected in anxiety disorders and depressive disorders, or problems experienced at the level of neurotransmitters (especially noradrenaline and dopamine) may influence pseudomyopia. Of course, this neurobiological explanation is just a hypothesis. However, the neuroanatomical structure of the near vision reflex has not been well studied in the human visual system. Lindberg, stressed that it is a functional disease triggered by long-term work and stress, and that this is inevitable.^20^ However, this information has not been confirmed in other studies.^16^

SNR spectrum, based on neurological etiology can be considered as acquired SNR and is based on the influences in the three neuroanatomic sites involving the cerebral cortex, the pretectum of the middle brain and the oculomotor nuclear complex.^21^ Although there was no positive family history for pseudomyopia, it was detected only in twin children without any etiology, which shows that it may be a disease with neurobiological basis.^22^

### Limitations of the study

Our study had a relatively low number of patients and that accommodative spasm was not evaluated after long-term follow-up of patients diagnosed with psychiatric disorders.

## CONCLUSION

Anxiety and depressive disorder can be admitted to the hospital with pseudomyopia. Because pseudomyopia may appear as a somatic symptom in internalizing disorders such as anxiety and depression. Patients may first consult an ophthalmologist. These psychiatric disorders can lead to pseudomyopia, because in cases of pseudomyopia, comorbidity of anxiety and depression are more common. Psychiatric evaluation is among the reasons to be considered in the etiology of pseudomyopia. It is necessary to request psychiatric consultation in each case of pseudomyopia since multidisciplinary treatment is needful for the follow-up of the disease. There is a need for studies to be performed with more patients and longtime follow-up.

### Authors’ Contribution:

**HK and EY** conceived idea.

**HK.** developed the theory.

**HK, EY and UG** worked equally during the data collection process.

**EY and UG** Data analysis

**HK** Accountable for the study.
